# Effect of prone positioning and PEEP on respiratory mechanics in children undergoing scoliosis surgery

**DOI:** 10.1186/s44158-025-00285-4

**Published:** 2025-10-15

**Authors:** Anna Camporesi, Federico Cristiani, Pablo Cruces, Horacio Igarzabal, Giulia Catozzi, Ginevra Bayon, Fernando Fontans, Gimena Falcao, Sofi Odriozola, Jurg Hammer, Sebastiàn Gonzalez-Dambrauskas

**Affiliations:** 1 Division of Pediatric Anesthesia and Intensive Care, Vittore Buzzi Children’s Hospital, Milan, Italy; 2 Department of Anesthesiology, Facultad de Medicina, Universidad de La República, Centro Hospitalario Pereira Rossell, Montevideo, Uruguay; 3 Laboratory of Translational Research in Critical Care, Center for Research On Pandemic Resilience, Faculty of Life Sciences, Universidad Andres Bello, Santiago, Chile; 4 Unidad de Paciente Crítico Pediátrico, Hospital El Carmen Dr. Luis Valentín Ferrada, Santiago, Chile; 5 Department of Pathophysiology and Transplantation, University of Milano, Milan, Italy; 6 Departamento de Pediatría y Unidad de Cuidados Intensivos de Niños del Centro Hospitalario Pereira Rossell, Facultad de Medicina, Universidad de La República, Montevideo, Uruguay; 7 Division of Respiratory and Critical Care Medicine, University Children’s Hospital Basel, University of Basel, Basel, Switzerland; 8 Red Colaborativa Pediátrica de Latinoamérica (LARed Network), Montevideo, Uruguay

**Keywords:** Scoliosis, Pediatrics, Mechanical ventilation, Prone position, Respiratory Mechanics, Positive end-expiratory pressure

## Abstract

**Background:**

Surgery for severe scoliosis (SS) is usually performed in the prone position. Changes in respiratory mechanics related to position and positive end expiratory pressure (PEEP) titration during anesthesia of SS are understudied.

We aimed to investigate the effect of prone position and PEEP on the respiratory mechanics of scoliotic children undergoing spine surgery.

**Methods:**

Prospective, crossover study performed in two pediatric hospitals (Montevideo, Uruguay-Centro Hospitalario Pereira Rossell- and Milano, Italy-Vittore Buzzi Children’s Hospital). Shortly after intubation, pulmonary mechanics measurements were performed using inspiratory and expiratory breath holds during volume-controlled ventilation with a set tidal volume (TV) of 8 ml/kg and a respiratory rate adjusted to maintain normocapnia. Measurements of peak (PIP), plateau (P_PLAT_) and total PEEP (tPEEP) were obtained at three levels of applied PEEP: 0 (ZEEP), 5, and 10 cmH_2_O both in supine (baseline) and prone positions. Driving pressure (∆P: P_PLAT_–tPEEP) was calculated to obtain static respiratory system compliance (Crs: TV/∆P). Crs and pressures were analyzed using a mixed linear regression model with a random subject effect in their relationship with position and PEEP.

**Results:**

Sixty-nine patients were enrolled. Crs was negatively associated with Cobb angle in all the cohorts. Only in secondary scoliosis, it was positively associated with body mass index. Crs was also negatively correlated with the prone position and positively correlated with increasing PEEP levels. The interaction between PEEP and position was studied and showed no significance.

**Conclusions:**

Crs is influenced by the severity of scoliosis and the nutritional status during spine surgery. The addition of PEEP improves Crs and reduces ∆P in the supine position, but both worsen in the prone position. These changes can be related to the effects of position on chest wall compliance.

## Key point summary

Question: This study investigates the effect of prone position and the application of PEEP on respiratory mechanics in children undergoing spinal surgery for severe scoliosis.

Findings: Respiratory system compliance was affected by the severity of scoliosis and the nutritional status, and it improved with application of PEEP, while decreased during prone position.

Meaning: Application on low-moderate PEEP level during scoliosis surgery is useful to counteract atelectasis formation due to lung compression following the primary disease and diaphragm elevation during prone position.

## Introduction

Scoliosis is a three-dimensional deformation of the spine that causes chest wall distortion, which induces a restrictive respiratory disease, in patients affected [1]. The restrictive pattern of respiratory disease in scoliosis arises from a reduction of lung volumes, impaired diaphragmatic excursion, and rib cage muscle inefficiency. This alters the mechanics of breathing and disables energy-efficient respiration [2, 3]. Scoliosis can be idiopathic or secondary to neuromuscular diseases (NMD) and surgery is sometimes required due to different reasons, ranging from psychological and mental well-being in idiopathic cases [4] to relief of severe pain [5] and stabilization of decline in respiratory function secondary scoliosis [6]. 

Spine surgery for scoliosis is a major surgical procedure [7] requiring, for most techniques, an intraoperative prone position. After intubation, a decrease in static respiratory system compliance (Crs) has been described in both adults [8] and pediatric patients [9], although the net effect of pronation is improved oxygenation. This position has been used for patients with severe acute respiratory distress syndrome (ARDS) since the 1970 s [10]. The addition of external positive end-expiratory pressure (PEEP) can improve Crs and elastic work of breathing by lung recruitment [11]. Concordantly, previous studies in healthy anesthetized children demonstrated that the addition of PEEP improves elastic work of breathing and Crs, probably by preventing end-expiratory alveolar collapse, recumbent atelectasis, and functional residual capacity (FRC) in the operating room [11]. However, there are no prior published data on how the change in position (from supine to prone) impacts the respiratory mechanics of children intubated for elective scoliosis surgery and how the mechanical properties change with the addition of PEEP [12].

The primary aim of the present study was to investigate the effect of prone position on respiratory mechanics in scoliotic children patients undergoing elective spine surgery. The secondary aim was to evaluate the mechanical effect of external PEEP in both positions.

## Methods

### Study design and setting

This prospective study was conducted between April 2023 and July 2024 in the operating rooms of two tertiary-level pediatric hospitals located respectively in Montevideo, Uruguay (Centro Hospitalario Pereira Rossell) and Milano, Italy (Vittore Buzzi Children’s Hospital). All procedures were followed in accordance with the ethical standards of the Helsinki Declaration of 1975. Ethical approval for this study was provided by the institutional review board of the two participating centers, and informed family consent and/or patient assent was obtained prior to the surgery (IRB approval nr: 7647719). A data analysis and statistical plan was written and filed with a private entity (IRB) before data were accessed. The manuscript follows the Strengthening the Reporting of Observational Studies in Epidemiology (STROBE) guidelines [13]. The study was unfunded.

### Participants

Children with severe scoliosis aged between 6 and 18 years, scheduled for elective spine surgery, were considered eligible and screened for the study. Patients were excluded if they lacked parental consent or if they had an acute illness or complication before (e.g., signs of acute lower respiratory tract infection) or during anesthesia (e.g., laryngospasm, bronchoconstriction, pneumothorax), previous thoracic surgery, airway malformation, chronic lung disease requiring long-term ventilation with oxygen dependence and/or tracheostomy. Additionally, patients with endotracheal tube air leak > 20% of tidal volume (TV) were excluded due to possible interference with data acquisition. Demographic and clinical data were anonymized and recorded in a previously designed Microsoft Excel® version 2021 Spreadsheet. Patients were categorized in two groups and classified as having primary scoliosis or secondary scoliosis according to the presence of a neuromuscular disease (NMD).

### Study protocol: procedures and respiratory mechanics measurements

Patients were monitored upon entrance into the operating room (OR) with standard monitoring: electrocardiogram (EKG), peripheral saturation of oxygen (SpO2), non-invasive blood pressure (NIBP), end-tidal capnography (etCO2), train-of-four (ToF), Bispectral Index (BIS). Anesthesia was induced with propofol, remifentanil, and rocuronium and maintained with a continuous infusion of propofol and remifentanil to sustain BIS values between 40 and 60. Neuromuscular blockade was monitored with ToF. Patients have been infused throughout surgery with balanced crystalloids (Sterofundin, BBraun) at 10 ml/kg/h to replace insensible losses. Acute blood losses were replaced with blood products. Tranexamic acid is administered in bolus (15 mg/kg) at skin incision and maintained in continuous infusion (3 mg/kg/h) up to skin closure.

Endotracheal tubes used were cuffed in all patients. Patients were ventilated with a Primus (Draeger, Luebeck, Germany) or a Maquet Flow-I (Maquet, Solna, Sweden) ventilator using the volume control mode (VCV) after intubation, verification of deep sedation, muscular paralysis, and confirming the correct positioning of the endotracheal tube. Baseline settings in the supine position were as follows: TV = 8 mL·kg^−1^, PEEP = 5 cmH_2_O, inspiratory:expiratory ratio = 1:2, and respiratory rate (RR) adjusted to achieve an end-tidal carbon dioxide (ETCO_2_) of 40 ± 5 mmHg. Tracheal tube leak compensation was deactivated during the measurements. Fraction of inspired oxygen (FiO_2_) was held at 0.4 during induction and maintenance.

Respiratory mechanics were measured in static conditions at the Y piece (proximal flow sensor), after an inspiratory hold maneuver followed by an expiratory hold maneuver (3–5 s each). The following variables were then measured: peak inspiratory pressure (PIP), plateau pressure (P_PLAT_), extrinsic (set) positive end-expiratory pressure (PEEP), total PEEP (tPEEP), intrinsic PEEP (tPEEP – PEEP), driving pressure (ΔP = P_PLAT_ – tPEEP), delivered TV (mL·kg^–1^), inspiratory time (IT). Data were registered for calculations of derived variables, like Crs (Crs: TV/ΔP, mL·cmH_2_O^–1^·kg^–1^) and dynamic compliance (Cdyn: TV/PIP – PEEP).

All the variables were subsequently normalized, adjusting TV by IBW [14]. IBW was determined by World Health Organization weight-for-length data, determining the z score, and assigning the median weight [15].

Before pronation for spinal surgery, we measured respiratory mechanics at three different PEEP levels: 0 (ZEEP), PEEP 5, and 10 cmH_2_O, in random order, separated by 5 min each to achieve stability, following our local protocol for respiratory mechanics measurements.

Patients were then turned prone, and the same three PEEP level measurements were repeated before surgical manipulation started. Cobb angle degree was defined as the angle between two lines drawn perpendicular to the upper endplate of the uppermost vertebra and the lower endplate of the lowest vertebra involved in the scoliosis curve [16].

### Statistical analysis

Results are expressed as *N* (%) for categorical measures and mean (SD) or median (IQR) as appropriate in case of continuous variables. Crs and Cdyn were tested for relationship with the BMI and the Cobb angle degree with univariate linear regression. The effect of PEEP and position on Crs, Cdyn, PIP, P_PLAT_ and ∆P were tested with multilevel mixed linear regression with a randomly varying subject effect.

Data were analyzed with Stata 18.0 B.E. (StataCorp LLC, USA) and R (R Core team. A Language and Environment for Statistical Computing. R Foundation for Statistical Computing, Vienna, Austria). All statistical tests were two-sided, and the level of statistical significance was set at 0.05.

### Sample size

Sample size was estimated using pediatric respiratory mechanics data from Chiumello et al. [17]. In their cohort of sedated children without ARDS, the mean respiratory system compliance was approximately 0.7 ml/cmH₂O/kg**,** with a reported standard deviation of about 0.12 ml/cmH₂O/kg**.** For a representative body weight of 40 kg, which is a representative weight in the studied cohort, this corresponds to a mean compliance of ≈ 28 ml/cmH₂O and an SD of ≈ 4.8 ml/cmH₂O in absolute units. Assuming a paired (within-subject) comparison between PEEP levels (e.g., 0 vs 10), two-sided α = 0.05 and 80% power, and adopting a conservative within-subject correlation of *r* = 0.30, the SD of the within-subject difference is ≈5.68 ml/cmH₂O. Under these assumptions, to detect a minimal clinically important difference of 2.0 ml/cmH₂O requires ≈64 subjects.

## Results

### Study cohort and characteristics

During the study period, 69 patients met inclusion criteria and were included in the final data analysis. Demographic data and ventilatory parameters of patients were reported in Table 1. No patient was excluded. There was no missing data.

**Table 1:** Demographic characteristics of the cohort

	Total	Primary scoliosis	Secondary scoliosis	*P*-value
	*N* = 69	*N* = 33	*N* = 36	
Age, years	13.0 (10.0–15.0)	14.0 (13.0–16.0)	12.0 (8.0–14.0)	< 0.001
Male sex	17 (25%)	4 (11%)	14 (37%)	< 0.001
Weight, kg	38.0 (24.0–51.0)	45.5 (39.0–59.0)	26.6 (18.0–35.0)	< 0.001
Ideal weight, kg	43.0 (27.0–47.0)	46.5 (43.0–49.5)	33.0 (23.0–42.0)	< 0.001
Height, m	1.5 (1.3–1.6)	1.6 (1.5–1.6)	1.4 (1.1–1.5)	< 0.001
BMI, kg/m^2^	16.9 (13.9–20.2)	19.0 (15.8–23.7)	14.5 (13.1–18.5)	< 0.001
Cobb degree, °	70.0 (62.0–81.0)	70.0 (60.0–79.0)	72.0 (63.0–89.0)	0.031
FEV1, % of predicted	0.6 (0.4–0.8)	0.8 (0.8–0.9)	0.4 (0.4–0.6)	< 0.001
FVC, % of predicted	0.6 (0.3–0.9)	0.8 (0.8–0.9)	0.4 (0.3–0.6)	0.001

Data are presented as *N*(%) or median (IQR)

*BMI *body mass index, *FEV1 *forced expiratory volume in 1 s, *FVC* forced vital capacity

Twenty-five percent were male, the median age was 13 years (10–15), and weight was 38 kg (24–51). 48% of the cohort was primary and 51% secondary scoliosis. Patients with secondary scoliosis included spinal muscular atrophy type 2 and 3, congenital muscular dystrophy, and nemalinic dystrophy.

**Fig. 1:**
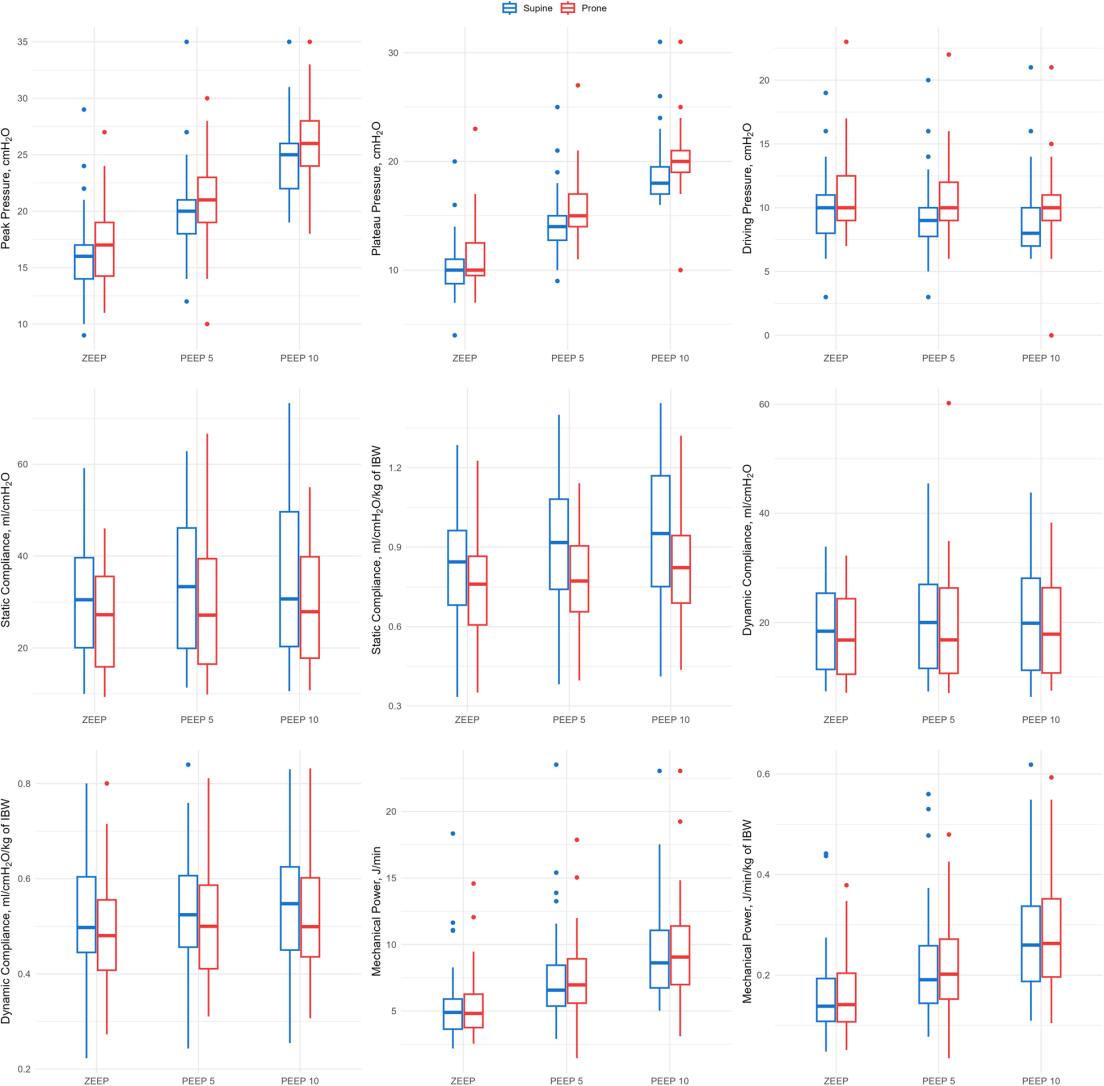
Measures of respiratory mechanics according to position and PEEP Level. PEEP: positive end-expiratory pressure

Respiratory measurements at the different steps of the study are reported in Table 2 and Figure 1.

**Table 2:** Respiratory mechanics across the different timepoints of the study

	Supine ZEEP	Supine PEEP 5	Supine PEEP 10	Prone ZEEP	Prone PEEP 5	Prone PEEP 10
PIP, cmH_2_O	16 (14–17)	20 (18–21)	25 (22–26)	17(14–19)	21 (19–23)	26 (24–28)
P_PLAT_, cmH_2_O	10 (8.5–11)	14 (12.5–15)	18 (17–20)	10 (9–13)	15 (14–17)	20 (19–21)
ΔP, cmH_2_O	10 (8–11)	9 (7.5–10)	8 (7–10)	10 (9–13)	10 (9–12)	10 (9–11)
Crs, ml/cmH_2_O/kg	0.85 (0.68–0.97)	0.92 (0.73–1.09)	0.95 (0.75–1.18)	0.76 (0.60–0.86)	0.77 (0.65–0.92)	0.82 (0.66–0.96)
Cdyn, ml/cmH_2_O/kg	0.50 (0.44–0.61)	0.55 (0.46–0.64)	0.57 (0.46–0.63)	0.48 (0.40–0.56)	0.50 (0.41–0.59)	0.51 (0.43–0.60)
FiO2	0.4 (0.4–0.4)	0.4 (0.4–0.4)	0.4 (0.4–0.4)	0.4 (0.4–0.4)	0.4 (0.4–0.4)	0.4 (0.4–0.4)

*Cdyn *dynamic respiratory system compliance, *Crs *static respiratory system compliance, *PEEP *positive end-expiratory pressure, *PIP *peak inspiratory pressure, *P*_*PLAT*_ plateau pressure. *ZEEP *zero end-expiratory pressure, *ΔP *driving pressure

### Respiratory mechanics and severity of scoliosis

In univariate analysis, Crs was positively associated with body mass index (BMI) (Coeff 0.01; 95% CI 0.003; 0.017; *P* = 0.006) in the secondary scoliosis subgroup but not in the primary scoliosis one. When adjusted for the Cobb angle, Crs was positively associated with BMI in all populations (Coeff 0.01; 95% CI 0.003; 0.017; *P* = 0.004). All respiratory mechanics’ measures were significantly correlated with Cobb angle degree of the scoliotic curvature. Relative coefficients are reported in Table 3.

**Table 3:** Relationship between measures of respiratory mechanics and Cobb angle degree

	Cobb angle		
	Coeff	95% CI	*P* value
PIP, cmH_2_O	0.04	0.007; 0.0817	0.018
P_PLAT_, cmH_2_O	0.04	0.009; 0.077	0.012
ΔP, cmH_2_O	0.04	0.024; 0.066	< 0.001
Crs, ml/cmH_2_O/kg	− 0.003	− 0.005; − 0.001	< 0.001
Cdyn, ml/cmH_2_O/kg	− 0.002	− 0.003; − 0.0008	0.001

*Cdyn *dynamic respiratory system compliance, *Crs* static respiratory system compliance, *PIP* peak inspiratory pressure, *P*_*PLAT*_ plateau pressure, *ΔP* driving pressure

### Effects of position and PEEP levels on respiratory mechanics

**Fig. 2:**
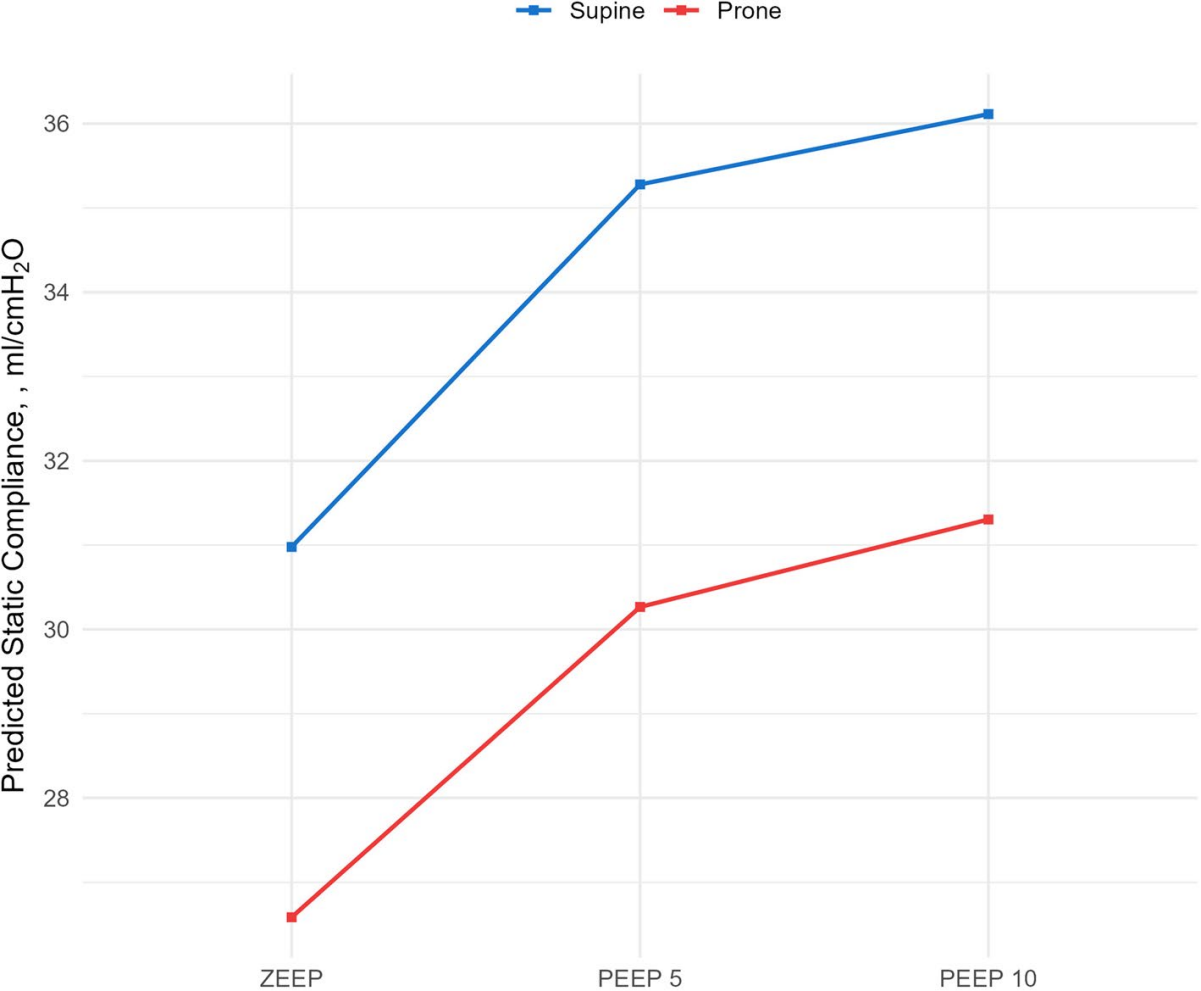
Predictive margins of Crs according to PEEP and position. Crs: static respiratory system compliance. PEEP: positive end-expiratory pressure

Respiratory mechanics was studied in its relationships with PEEP and position and presence of a comorbidity with multilevel mixed linear regression including a random effect on the subjects (Table 4). Crs was negatively correlated with prone position and positively correlated with increasing PEEP levels (Fig. 2). The interaction between PEEP and position was studied and showed no significance on Crs. ΔP was reduced by PEEP and increased in prone position.

**Table 4:** Mixed linear regression outcomes for Crs and different PEEP levels and supine vs prone position

	Crs			Cdyn			PIP			P_PLAT_			ΔP		
	Coeff	*P* values	95% CI	Coeff	*P* values	95% CI	Coeff	*P* values	95% CI	Coeff	*P* values	95% CI	Coeff	*P* values	95% CI
Position															
Prone	− 0.14	< 0.001	− 0.17; − 0.10	− 0.04	< 0.001	− 0.06; − 0.02	1.47	< 0.001	1.11;1.83	1.35	< 0.001	1.05;1.64	1.31	< 0.001	1.01; 1.62
PEEP (cmH_2_O)															
5	0.06	0.001	0.02; 0.10	0.044	< 0.001	0.02; 0.06	3.84	< 0.001	3.42;4.26	4.03	< 0.001	3.68; 4.38	−0.84	< 0.001	− 1.20; − 0.49
10	0.08	< 0.001	0.04; 0.13	0.049	< 0.001	0.02; 0.07	8.38	< 0.001	7.92;8.83	8.42	< 0.001	8.05–8.80	−1.46	< 0.001	− 1.85; − 1.08
Comorb	− 0.03	0.488	− 0.15; 0.07	− 0.07	0.064	− 0.14; 0.02	1.87	0.009	0.46; 3.27	0.63	0.184	− 0.30; 1.58	0.59	0.223	− 0.36; 1.55

Prone position coefficient is referred to supine position; PEEP 5 and 10 are referred to ZEEP. *Crs* static respiratory system compliance, *PEEP *positive end-expiratory pressure*, PIP* peak inspiratory pressure, *P*_*PLAT*_ plateau pressure, *ZEEP* zero end-expiratory pressure, *ΔP* driving pressure

## Discussion

We studied the effect of position and incremental PEEP levels on respiratory mechanics in anesthetized children with severe scoliosis undergoing scoliosis surgery, and our main finding is that Crs is influenced by the severity of the scoliosis (expressed by Cobb angles), the nutritional status, the set PEEP, and the body position.

### Severity of scoliosis

We identified that the severity of scoliosis, described by the degree of the Cobb angle, showed a negative linear relationship with Crs. Concordantly, reduced Crs has been reported in adults with severe kyphoscoliosis [18], and there is an inverse correlation between its severity and respiratory function in adolescents [19]. This could be explained by the fact that the curvature of the spine is three-dimensional; therefore, it leads to a rotation of the vertebrae and the rib cage and a reduction in the sagittal diameter of the thorax. This reduces the space allocated for the lungs, compresses the lung itself, and reduces the chest wall compliance (Ccw). Interestingly, the presence of NMD did not affect the relationship between Crs and the Cobb angle, suggesting that the effect of scoliosis is entirely due to the mechanical effect of rotation and not by muscle fibrosis or stiffness.

### Nutritional status

Crs was also positively associated with BMI in secondary scoliosis, but not in primary scoliosis. It is noteworthy that our population presented unusually low BMIs in both primary and secondary scoliosis, being more pronounced in the latter. Children with secondary scoliosis frequently have a nutritional impairment [20], although those with primary scoliosis also tend to have low BMIs. Low nutritional status and scoliosis are closely interrelated: a low BMI leads to reduced strength in the paraspinal muscles, which may trigger scoliosis in the long term, but scoliosis can also lead to a rejection of body acceptance and anorexia [21].

In the secondary scoliosis subgroup, the positive relation between Crs and BMI probably expresses the fact that lower BMIs are a proxy for the severity of the primary disease and thus for the stiffness of the rib cage.

Altogether, the correlation of Crs with Cobb angle and BMI supports that Ccw rather than lung compliance is the major contributor to the total Crs. The decrease in Crs might be expected mainly due to their stiffer chest wall. Unfortunately, we did not use an esophageal manometry, and Ccw could not be measured to confirm this hypothesis.

### Set PEEP

Respiratory mechanics were also significantly modified by PEEP and position. In the supine position, there was a clear improvement in Crs from 0 to 5 cmH_2_O of PEEP. The observed decrease in ∆P with the addition of PEEP was previously reported by our group, although the studied cohort was healthy anesthetized children [11]. PEEP has been postulated to improve Crs by the reduction of atelectatic areas of the lung [22, 23]. Atelectasis is common during anesthesia due to multiple reasons: neuromuscular blockade causing a reduction in FRC by lack of respiratory muscle activity, compression of small airways by intrathoracic organs such as the heart, and increased pleural pressure [24]. PEEP can prevent and reverse atelectasis and therefore improve Crs. The lack of improvement in Crs with PEEP 10 cmH₂O was consistently observed across the study cohort, regardless of the random order in which PEEP levels were applied. This suggests that the observed plateau effect represents a true physiological limit rather than a methodological artifact.

We hypothesize that the transition from ZEEP to 5 cmH_2_O PEEP restores the pre-anesthesia FRC as previously shown by Von Ungern-Sternberg et al. [25], while the subsequent addition of 5 more cmH_2_O PEEP leads the system to a flatter portion of the pressure–volume curve. PEEP at 5 cmH_2_O is possibly a good compromise between a predominantly collapsed (ZEEP) and overinflated zone (PEEP 10 cmH_2_O) [26]. The physics of the lung inflated by a single pressure waveform results in unpredictable gas distribution due to variable regional compliances [27]. Crs is an overall measure that may provide some clues about lung recruitment but does not provide regional information. Moreover, although the TV (indexed by IBW) used for the measurements was reasonable, we could hypothesize that it was too big for such rib cages where lungs are chronically rotated and compressed [3]. The increase in Crs after adding PEEP is most likely the result of recruitment of collapsed alveoli, since FRC is even lower in these children.

### Elastic changes during prone position

We also detected a reduction in Crs in the prone position. There are prior physiological reports which can help explain how the prone position works during invasive mechanical ventilation (IMV) in the operating room. During pronation, changes in respiratory mechanics occur, both in spontaneously breathing patients [28] and in mechanically ventilated ones [8]. 

As the prone position limits the movement of the rib cage and can push the diaphragm upwards through increased pressure on the free wall of the abdomen, the reduction in Crs is possibly attributable to the reduction of the chest wall component.

There are contradictory reports regarding the mechanical effects of the prone position in healthy anesthetized adults. Pelosi et al. did not find any significant changes in Crs, Ccw, and lung compliance [8]. Conversely, Tanskanen et al. found a decrease in dynamic compliance [29]. During supine, atelectasis usually occurs in dependent lung tissue, and when turned prone, these atelectatic regions become the non-dependent ones and could be reopened. Since the spine surgery bed is customized with four support pads for the patient’s thorax, leaving the abdomen relatively free to move, and limiting the movement of the anterior part of the chest wall, anterior Ccw is reduced. However, our cohort probably presents a reduction in Ccw, which potentially limits posterior movement while prone, thus making it not possible to re-open these areas, and this, together with the anterior limitation in the prone position, could explain a reduced Crs.

Prone position was introduced in clinical practice during the 1970 s after the pediatrician AC Bryan published the theoretical background for its use to improve respiratory function during IMV [30]. In 1976, MA Piehl and RS Brown reported for the first time marked improvement in oxygenation during IMV in the prone position in 5 patients with acute lung injury (two of which were children) [31]. Currently, the prone position is a frequent rescue therapy in ARDS for both adults and children [10, 32]. The dichotomy between worse Crs and better oxygenation has a physiological explanation. During the prone position, there is less gravitational impact of the mediastinum weight on the lungs, while pulmonary perfusion is preserved, improving the ventilation–perfusion coupling and oxygenation. In accordance, von Ungern-Sternberg found that the prone position during general anesthesia improves FRC and volume distribution in healthy paralyzed children [12]. In our study, PEEP did not significantly increase Crs in the prone position, and it even decreased when PEEP was elevated to 10 cmH_2_O, suggesting overdistention. Our results differ from those of Spaeth, who found that raising PEEP levels from 6 to 9 cmH_2_O in the prone position increased Crs and aeration in obese adults without scoliosis [33]. We can speculate that obesity superimposes a gravitational pressure on the lungs, increasing the collapse and reducing the Crs. In this context, PEEP reverses collapse and improves Crs.

### Limitations and strengths

We acknowledge our study presents some limitations. First, as we did not measure esophageal pressures, we could not discriminate between chest wall and lung compliances, which could have helped to explain how much of the Crs is contributed by atelectasis and could benefit from PEEP addition. Second, we did not measure lung perfusion nor aeration, so we were not able to evaluate how changes during prone ventilation could alter FRC and other important determinants of oxygenation. Third, during prone position, the highest level of PEEP applied in our cohort was 10 cmH_2_O. A recent study in healthy infants during surgery found that higher PEEP levels could improve lung aeration and ventilation distribution [34]. Our study, however, included the largest cohort of children operated on for severe scoliosis to our knowledge and provides important data for anesthesiologists. Finally, although the results of our study may not be easily extrapolated to cohorts of children without scoliotic disease, our data provide important physiological information for applying the prone position in other groups of children undergoing IMV. Future studies will have to investigate these outcomes and what is the best method to titrate PEEP levels in children with and without scoliosis during surgery.

### Clinical implications and conclusions

We demonstrated that Crs is influenced by Cobb angles during scoliosis surgery. While the addition of PEEP improves the Crs and reduces ΔP in the supine position, deterioration of Crs during prone ventilation is predictable intraoperatively. The addition of low-moderate PEEP levels during scoliosis surgery is important, and although the exact titration of the optimal level should continue to be studied, it is advisable to perform adequate monitoring of respiratory mechanics to detect unnoticed deteriorations. During prone position, Crs might not benefit from PEEP levels beyond 10 cmH_2_O. The use of a PEEP around 5 cmH_2_O is probably more physiological and enough to avoid atelectasis.

## Data Availability

No datasets were generated or analysed during the current study.

## References

[1] Redding G, Song K, Inscore S, Effmann E, Campbell R (2008) Lung function asymmetry in children with congenital and infantile scoliosis. Spine J 8(4):639–644. 10.1016/j.spinee.2007.04.020 17923444 10.1016/j.spinee.2007.04.020

[2] Kato S, Murray JC, Ganau M, Tan Y, Oshima Y, Tanaka S (2019) Does posterior scoliosis correction improve respiratory function in adolescent idiopathic scoliosis? A systematic review and meta-analysis. Glob Spine J 9(8):866–873. 10.1177/2192568218811312 10.1177/2192568218811312 PMC6882091 31819853

[3] Koumbourlis AC (2006) Scoliosis and the respiratory system. Paediatr Respir Rev 7(2):152–160. 10.1016/j.prrv.2006.04.009 16765303 10.1016/j.prrv.2006.04.009

[4] Agabegi SS, Kazemi N, Sturm PF, Mehlman CT (2015) Natural history of adolescent idiopathic scoliosis in skeletally mature patients: a critical review. J Am Acad Orthop Surg 23(12):714–723. 10.5435/JAAOS-D-14-00037 26510624 10.5435/JAAOS-D-14-00037

[5] Miyanji F, Nasto LA, Sponseller PD (2018) Assessing the risk-benefit ratio of scoliosis surgery in cerebral palsy: surgery is worth it. J Bone Joint Surg 100(7):556–563. 10.2106/JBJS.17.00621 29613924 10.2106/JBJS.17.00621

[6] Chou SH, Lin GT, Shen PC et al (2017) The effect of scoliosis surgery on pulmonary function in spinal muscular atrophy type II patients. Eur Spine J 26(6):1721–1731. 10.1007/s00586-016-4828-2 27807779 10.1007/s00586-016-4828-2

[7] Basques BA, Lukasiewicz AM, Samuel AM (2017) Which pediatric orthopaedic procedures have the greatest risk of adverse outcomes? J Pediatr Orthop 37(6):429–434. 10.1097/BPO.0000000000000683 26558959 10.1097/BPO.0000000000000683

[8] Pelosi P, Croci M, Calappi E (1995) The prone positioning during general anesthesia minimally affects respiratory mechanics while improving functional residual capacity and increasing oxygen tension. Anesth Analg 80(5):955–960. 10.1097/00000539-199505000-00017 7726438 10.1097/00000539-199505000-00017

[9] Cox RG, Ewen A, Bart BB (2001) The prone position is associated with a decrease in respiratory system compliance in healthy anaesthetized infants. Pediatr Anesth 11(3):291–296. 10.1046/j.1460-9592.2001.00646.x 10.1046/j.1460-9592.2001.00646.x 11359586

[10] Sud S, Friedrich JO, Adhikari NKJ et al (2014) Effect of prone positioning during mechanical ventilation on mortality among patients with acute respiratory distress syndrome: a systematic review and meta-analysis. CMAJ 186(10):E381–E390. 10.1503/cmaj.140081 24863923 10.1503/cmaj.140081 PMC4081236

[11] Cruces P, González-Dambrauskas S, Cristiani F et al (2018) Positive end-expiratory pressure improves elastic working pressure in anesthetized children. BMC Anesthesiol 18(1):151. 10.1186/s12871-018-0611-8 30355345 10.1186/s12871-018-0611-8 PMC6201576

[12] Von Ungern-Sternberg BS, Hammer J, Frei FJ, Jordi Ritz EM, Schibler A, Erb TO (2007) Prone equals prone? Impact of positioning techniques on respiratory function in anesthetized and paralyzed healthy children. Intensive Care Med 33(10):1771–1777. 10.1007/s00134-007-0670-7 17558496 10.1007/s00134-007-0670-7

[13] von Elm E, Altman DG, Egger M, Pocock SJ, Gøtzsche PC, Vandenbroucke JP (2007) The strengthening the reporting of observational studies in epidemiology (STROBE) statement: guidelines for reporting observational studies. Lancet 370(9596):1453–1457. 10.1016/S0140-6736(07)61602-X 18064739 10.1016/S0140-6736(07)61602-X

[14] Emeriaud G, López-Fernández YM, Iyer NP (2023) Executive summary of the second international guidelines for the diagnosis and management of Pediatric Acute Respiratory Distress Syndrome (PALICC-2). Pediatr Crit Care Med 24(2):143–168. 10.1097/PCC.0000000000003147 36661420 10.1097/PCC.0000000000003147 PMC9848214

[15] Growth reference 5–19 years - BMI-for-age (5–19 years) https://www.who.int/tools/growth-reference-data-for-5to19-years/indicators/bmi-for-age. Accessed 11 September 2024

[16] Brodeur R, Menke JM, Elbert R, Brodeur RR, Menke JM, Elbert RA (2012) Scoliosis, chapter 13 of pediatric chiropractic, Anrig and Plaugher Editors 629–668

[17] Chiumello D, Chidini G, Calderini E, Colombo A, Crimella F, Brioni M (2016) Respiratory mechanics and lung stress/strain in children with acute respiratory distress syndrome. Ann Intensive Care 6(1):11. 10.1186/s13613-016-0113-0 26847436 10.1186/s13613-016-0113-0 PMC4742456

[18] Conti G, Rocco M, Antonelli M et al (1997) Respiratory system mechanics in the early phase of acute respiratory failure due to severe kyphoscoliosis. Intensive Care Med 23(5):539–544. 10.1007/s001340050370 9201526 10.1007/s001340050370

[19] Kempen DHR, Heemskerk JL, Kaçmaz G et al (2022) Pulmonary function in children and adolescents with untreated idiopathic scoliosis: a systematic review with meta-regression analysis. Spine J 22(7):1178–1190. 10.1016/j.spinee.2021.12.011 34963629 10.1016/j.spinee.2021.12.011

[20] Ferrantini G, Coratti G, Onesimo R et al (2022) Body mass index in type 2 spinal muscular atrophy: a longitudinal study. Eur J Pediatr 181(5):1923–1932. 10.1007/s00431-021-04325-3 35048179 10.1007/s00431-021-04325-3 PMC9056453

[21] Scaturro D, Balbo A, Vitagliani F, Stramazzo L, Camarda L, Letizia Mauro G (2022) Is there a relationship between idiopathic scoliosis and body mass? A scoping review. Nutrients 14(19):4011. 10.3390/nu14194011 36235665 10.3390/nu14194011 PMC9572444

[22] Cruces P, Reveco S, Caviedes P, Díaz F (2024) Respiratory system compliance accurately assesses the “baby lung” in pediatric acute respiratory distress syndrome. Am J Respir Crit Care Med 209(7):890–893. 10.1164/rccm.202310-1890LE 38324072 10.1164/rccm.202310-1890LE PMC10995576

[23] Roveri G, Camporesi A, Hofer A, Kahlen S, Breidt F, Rauch S (2025) Preoxygenation with and without positive end-expiratory pressure in lung-healthy volunteers: a randomized clinical trial. JAMA Netw Open 8(5):e2511569. 10.1001/jamanetworkopen.2025.11569 40392551 10.1001/jamanetworkopen.2025.11569 PMC12093187

[24] Zeng C, Lagier D, Lee JW, Vidal Melo MF (2022) Perioperative pulmonary atelectasis: Part I. biology and mechanisms. Anesthesiology 136(1):181–205. 10.1097/ALN.0000000000003943 34499087 10.1097/ALN.0000000000003943 PMC9869183

[25] Von Ungern-Sternberg BS, Hammer J, Schibler A, Frei FJ, Erb TO (2006) Decrease of functional residual capacity and ventilation homogeneity after neuromuscular blockade in anesthetized young infants and preschool children. Anesthesiology 105(4):670–675. 10.1097/00000542-200610000-00010 17006063 10.1097/00000542-200610000-00010

[26] Soni N, Williams P (2008) Positive pressure ventilation: what is the real cost? Br J Anaesth 101(4):446–457. 10.1093/bja/aen240 18782885 10.1093/bja/aen240

[27] Hurtado DE, Erranz B, Lillo F et al (2020) Progression of regional lung strain and heterogeneity in lung injury: assessing the evolution under spontaneous breathing and mechanical ventilation. Ann Intensive Care 10(1):107. 10.1186/s13613-020-00725-0 32761387 10.1186/s13613-020-00725-0 PMC7407426

[28] Moreno F, Lyons HA (1961) Effect of body posture on lung volumes. J Appl Physiol 16(1):27–29. 10.1152/jappl.1961.16.1.27 13772524 10.1152/jappl.1961.16.1.27

[29] Tanskanen P, Kyna J, Randell T (1997) The effect of patient positioning on dynamic lung compliance. Acta Anaesthesiol Scand 41(5):602–606. 10.1111/j.1399-6576.1997.tb04750.x 9181161 10.1111/j.1399-6576.1997.tb04750.x

[30] Bryan AC (1974) Conference on the scientific basis of respiratory therapy. Pulmonary physiotherapy in the pediatric age group. Comments of a devil’s advocate. Am Rev Respir Dis 110(6 Pt 2):143–144. 10.1164/arrd.1974.110.6P2.143 4440945 10.1164/arrd.1974.110.6P2.143

[31] Piehl MA, Brown RS (1976) Use of extreme position changes in acute respiratory failure. Crit Care Med 4(1):13–14. 10.1097/00003246-197601000-00003 1253612 10.1097/00003246-197601000-00003

[32] Qin W, Mao L, Shen Y, Zhao L (2024) Prone position in the mechanical ventilation of acute respiratory distress syndrome children: a systematic review and meta-analysis. Front Pediatr 12:1293453. 10.3389/fped.2024.1293453 38516357 10.3389/fped.2024.1293453 PMC10955119

[33] Spaeth J, Schumann S, Humphreys S, Ungern‐Sternberg B, ed (2022) Understanding pediatric ventilation in the operative setting. Part II: Setting perioperative ventilation. Pediatr Anesth 32(2):247–254. 10.1111/pan.14366 10.1111/pan.14366 34877746

[34] Lee J, Kang P, Park J (2024) Determination of optimal positive end‐expiratory pressure using electrical impedance tomography in infants under general anesthesia: comparison between supine and prone positions. Paediatr Anaesth. 10.1111/pan.14914 38693633 10.1111/pan.14914

